# Intrinsic organization of cortical networks predicts state anxiety: an functional near-infrared spectroscopy (fNIRS) study

**DOI:** 10.1038/s41398-020-01088-7

**Published:** 2020-11-20

**Authors:** Lian Duan, Nicholas T. Van Dam, Hui Ai, Pengfei Xu

**Affiliations:** 1 Shenzhen Key Laboratory of Affective and Social Neuroscience, Magnetic Resonance Imaging Center, Center for Brain Disorders and Cognitive Sciences, Shenzhen University, Shenzhen, China; 2 School of Psychological Sciences, University of Melbourne, Melbourne, Australia; 3 Department of Psychiatry, Icahn School of Medicine at Mount Sinai, New York, New York USA; 4 Beijing Key Laboratory of Applied Experimental Psychology, Faculty of Psychology, Beijing Normal University, Beijing, China; 5 Center for Neuroimaging, Shenzhen Institute of Neuroscience, Shenzhen, China; 6 Guangdong-Hong Kong-Macao Greater Bay Area Research Institute for Neuroscience and Neurotechnologies, Kwun Tong, Hong Kong, China

**Keywords:** Predictive markers, Human behaviour

## Abstract

Although state anxiety has been characterized by hyper-responsive subcortical activity and its bottom-up connectivity with cortical regions, the role of cortical networks in state anxiety is not yet well understood. To this end, we decoded individual state anxiety by using a machine-learning approach based on resting-state functional connectivity (RSFC) with functional near-infrared spectroscopy (fNIRS). Our results showed that the RSFC among a set of cortical networks were highly predictive of state anxiety, rather than trait anxiety. Specifically, these networks included connectivity between cortical areas in the default mode network (DMN) and dorsal attention network (DAN), and connectivity within the DMN, which were negatively correlated with state anxiety; connectivity between cortical areas in the DMN and frontoparietal network (FPN), FPN and salience network (SN), FPN and DAN, DMN and SN, which were positively correlated with state anxiety. These findings suggest a predictive role of intrinsic cortical organization in the assessment of state anxiety. The work provides new insights into potential neural mechanisms of emotion states and implications for prognosis, diagnosis, and treatment of affective disorders.

## Introduction

Anxiety characterizes a subjective emotional state, relating to spatially or temporally distant and/or uncertain threat; it is often accompanied by autonomic arousal and behavioral avoidance^1,2^. In contrast to fear which represents a more automated response to an imminent or immediate threat, anxiety is commonly associated with an individual’s apprehension about potential or distant harm/threat^2^. While *trait* anxiety reflects an individual’s predisposition for anxious responses, *state* anxiety reflects a temporary, subjective experience of apprehension about a potential threat or negative experience^3,4^. Thus, *state* anxiety represents a crucial situational relationship between physiological and subjective experiences of potential threat/harm^5^. State anxiety also reflects a potential precursor to broader tendencies in that the regular recurrence of particular states is likely to underpin the development of traits, over time^6,7^. While the veracity of subjective experience is, by definition, impossible to ascertain, objective markers of the subjective experience of fear may be critical to understanding why some people experience exaggerated threat responses while others do not^2^. To be sure, these exaggerations likely stem from some critical vulnerabilities (e.g., elevated trait anxiety or neuroticism) but having some way of easily tracking the individual’s state response as events are unfolding could hold much potential for predicting and treating anxiety disorders.

Although some studies suggest common brain networks among state, trait and pathological anxiety^8,9^, psychometric (and related brain imaging) analyses suggest that cognitive and somatic/physiological anxiety are quite different^1,10,11^. State anxiety has been shown to be more correlated with physiological/somatic symptoms than is trait anxiety^12^. Thus, state anxiety is more likely to reflect an individual’s subjective (bodily) attention to and/or interpretation of their present apprehension about imminent threat or harm, providing a key link between trait anxiety and the fear response. Indeed, recent work has shown that while trait anxiety tracks with intrinsic connectivity in midline cortical areas, state anxiety is more associated with activity in the insula^13^. Psychotherapeutic treatments for anxiety focus on reducing broader maladaptive behavioral patterns (consistent with trait anxiety) across treatment but also focus in session on experience at the moment, i.e., state anxiety; see, e.g., ref. ^14^.

On the basis of neuroimaging studies for brain responses of anxious individuals to attentional^15^ and emotional^16,17^ processes, previous models highlight the role of hyperactive bottom-up input of the amygdala during specific processes in state anxiety^18,19^. However, recent a brain network model^20^ and meta-analysis^9^ have shown common connectivity patterns within and between various cortical areas between anxiety and anxiety disorders^21^, including alterations in the frontoparietal control network (FPN), default mode network (DMN), dorsal attention network (DAN), ventral attention network, salience network (SN), and sensorimotor network. Interestingly, sustainedly altered activity in the vmPFC of the DMN has been observed during temporally persistent states of anxiety regardless of emotional manipulations^22^, pointing to alterations of intrinsic activity in state anxiety.

Resting-state functional connectivity (RSFC) is assumed to characterize commonly intrinsic representations of functional brain architecture across cognitive functions and is predictive of brain responses to task manipulations^23^ and individual difference^24^. While a recent proposed connectome-based predictive modeling model has been shown to effectively detect cognitive and brain state variability based on features from RSFC with full cross-validation^25,26^, both structural^27^ and functional^28^ connectivity of limbic areas with prefrontal regions have been shown to predict individual variations in trait anxiety. Individual differences in functional connectivity have been shown to be composed of both trait-relevant and state-dependent characteristics^29^. However, whether state anxiety could be predictable based on the intrinsic connectivity of brain networks, especially the cortical networks remain unclear. To this end, we conducted a functional near-infrared spectroscopy (fNIRS) study in individuals with various levels of state anxiety. We expected that the cortical areas in the previously proposed networks such as FPN and DMN should be able to predict state anxiety.

## Materials and methods

### Participants and paradigm

Ninety-six healthy young adults (22.8 ± 2.7 years of age, 48 female) participated. The sample sizes were determined based on previous studies about the connectivity-based prediction of anxiety^27,28^. No participants had any history of psychiatric illness or neurological disease. All provided informed consent prior to the experiment. The study protocol was approved by the Institutional Review Board at Shenzhen University.

All participants underwent a 7-min session of resting-state fNIRS recording. They sat in a comfortable chair with their eyes closed and were instructed to keep still, to relax their mind, to remain awake, and not to think about anything systematically^30^. No participants reported sleep or were observed to be sleeping during fNIRS recording. After fNIRS data collection, participants completed the state and trait versions of the State-Trait Anxiety Inventory^31^.

### fNIRS data acquisition and preprocessing

**Fig. 1:**
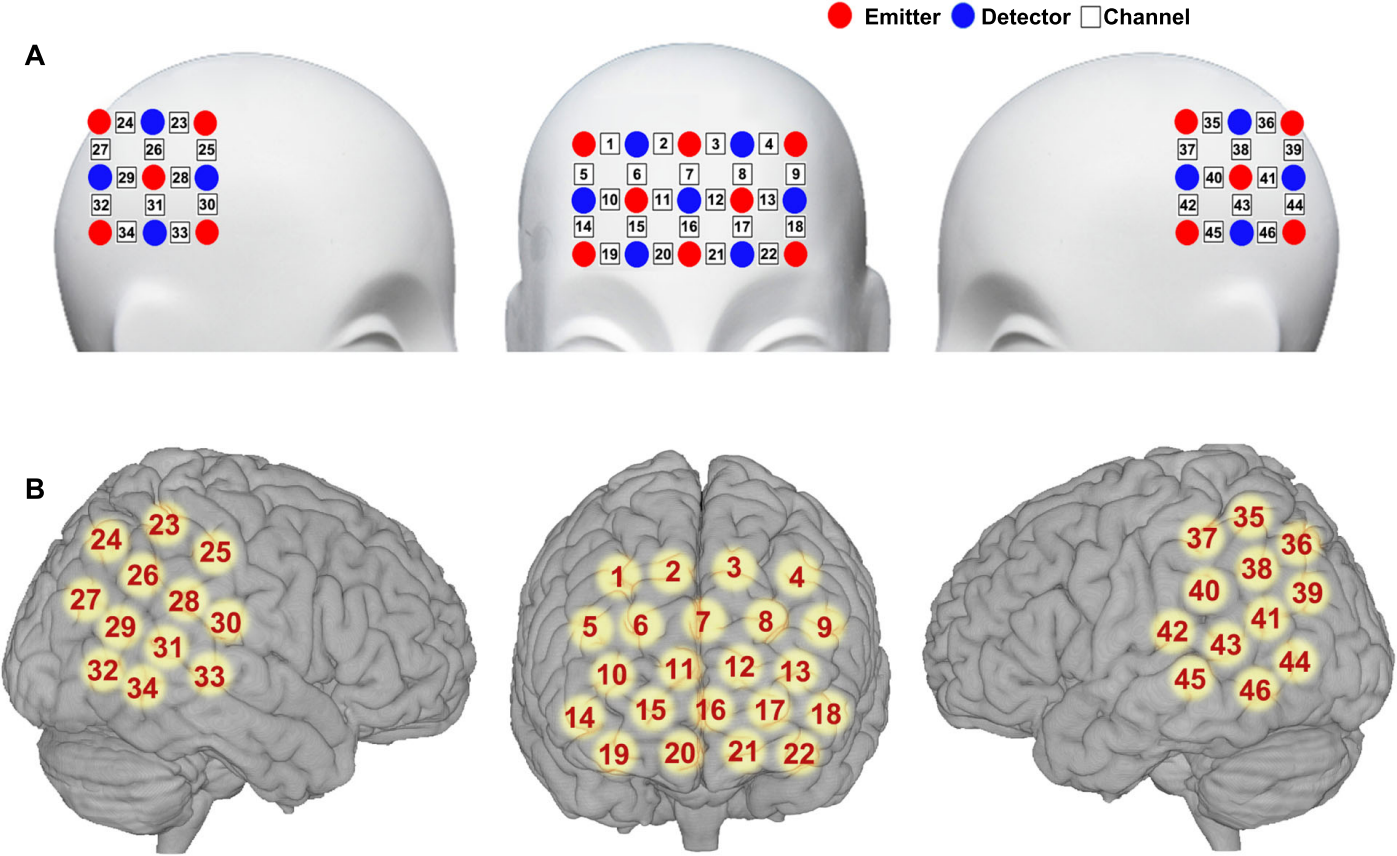
Schematic representations of optodes and channels. **A** The configuration of the fNIRS probes and **B** the localization of the fNIRS measurement channels.

The fNIRS measurement was conducted with an ETG-4000 continuous-wave optical topography system (Hitachi Medical Company, Tokyo, Japan). The absorption of the near-infrared light at two wavelengths (695 and 830 nm) was measured with a sampling rate of 10 Hz. Three pieces of probe sets were used, including one-piece placed on the frontal area and two pieces placed on the bilateral temporal-parietal areas, forming 46 channels in total. The frontal probe set was placed by approximately putting its bottom middle optode on Fpz of the international 10–20 system^32^, and the bilateral temporal-parietal probe sets were placed by approximately putting their anterior inferior optode on T7 and T8, respectively (Fig. 1). The source-detector distance was 30 mm. The cortical localizing MNI coordinates of the channels were obtained by using a 3-dimensional digitizer and the NIRS-SPM software^33,34^.

The light absorption data were converted to oxygenated (HbO) and deoxygenated (HbR) hemoglobin data via application of the modified Beer–Lambert law^35^ with a differential pathlength factor of 6.26^36^. The first and the last 10 s of data were discarded to ensure steady-state. Data were visually inspected to reject artifacts such as motion-related noises^37,38^. To remove superficial physiological noise and its related spurious connectivity^39,40^, the wavelet-based method was used to remove global physiological noise from the signal^41^. The data were band-pass filtered (0.01–0.08 Hz) to extract spontaneous neural activity^42^.

### Feature extraction

To extract features for prediction of individual phenotypic differences, we defined the fNIRS channels as nodes within specific resting-state networks cf^43^. RSFC was calculated between each channel and all other channels, representing network edges, resulting in a resting-state network containing 46 nodes and 1035 edges for each participant. We defined the RSFC as the Pearson correlation coefficient between the time courses of each pair of channels^44^. All 1035 RSFC values made up the feature vector which was used to predict individual phenotypic variation.

### Prediction model

**Fig. 2:**
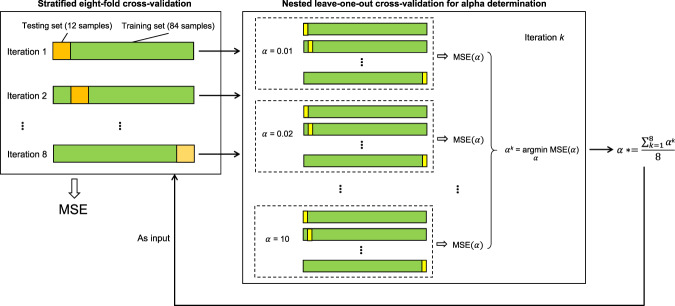
Schematic of the model fit. To fit the predictive model, we used a stratified eightfold cross-validation approach. We performed eight iterations of model training and testing. To determine the regularization parameter alpha, the training set of each iteration was entered into a nested leave-one-out cross-validation process. Specifically, in iteration *k* (*k* = 1, 2,⋯, 8), we traversed the alpha values (*α*) from 0.01 to 10 in steps of 0.01. For each *α*, we conducted a leave-one-out cross-validation and calculated the corresponding mean squared error, MSE(*α*). Then we picked the *α* which minimized MSE(*α*), denoted as *α*^*k*^. We averaged *α*^*k*^ (*k* = 1, 2,⋯, 8) and obtained a mean value α^*^, which was used as the regularization parameter for model training.

We built a multiple linear prediction models based on ridge regression (Fig. 2). Ridge regression is a regularized regression model which is particularly effective when the number of predictors is much bigger than the number of observations and collinearity exists among the predictors^45^. To assess whether the regression model could predict the individual differences in anxiety, we used a stratified eightfold cross-validation approach with the nested cross-validation for the regularization parameter estimation^27^. Specifically, we split the data of 96 participants eightfold. Each fold consisted of 12 participants and had a similarly distributed range of anxiety values. We performed eight iterations of model training and testing. In the *k*th iteration, we kept the *k*th fold of data as the testing set (12 samples) and used the remaining sevenfold of data as the training set (84 samples) to train the regression model. To determine the regularization parameter (alpha) of the ridge regression, for every iteration, we traversed the alpha values from 0.01 to 10 in steps of 0.01. For every alpha value, we conducted a leave-one-out cross-validation (LOOCV) only using the training set. We averaged the alpha values corresponding to the maximum mean accuracy of the LOOCV across iteration to obtain a mean alpha value for model training. The mean alpha value was also used in a permutation test (see below). We tested the model of each iteration using the reserved testing set and produced 12 predicted anxiety scores. We concatenated the predicted anxiety scores from all the eight iterations and obtained a vector of 96 predicted anxiety scores, one predicted score for each participant. We calculated the accuracy of the predictions by computing the mean squared error (MSE) and the Pearson correlation coefficient (*r*) between the predicted and actual state/trait anxiety scores.

To test the statistical significance of the accuracy of the predictions, we conducted a permutation test by randomly pairing the samples and the anxiety scores. We performed 10,000 permutations and calculated the MSE of the model fit. We determined the *p* value as the proportion of iterations in which the MSE derived from the randomized data were smaller than or equal to that derived from the real data.

To validate the model performance, we also estimated the model by using different cross-validation schemes (4, 6, 12, 16-fold), and calculated the correlation coefficients between actual and predicted anxiety scores.

### RSFC in the prediction of anxiety

In order to determine which features (i.e., which resting-state functional connectivity) significantly contributed to the prediction of the individual differences in anxiety, we used a bootstrap approach as used in previous studies^27,46^. Specifically, we performed 1000 bootstrap samplings with replacement. We generated 1000 independent regression models and estimated the 99% confidence interval of each feature’s weight. Those features whose 99% confidence interval was either entirely above or below zero were determined as significantly contributing features. To further characterize the connectivity properties of significant RSFC, we determined the anatomical localization and the resting-state network affiliation of terminal regions by using the automated anatomical labeling template^47^ and the seven-network brain surface parcellation template^48^, respectively.

## Results

### Prediction model

**Fig. 3:**
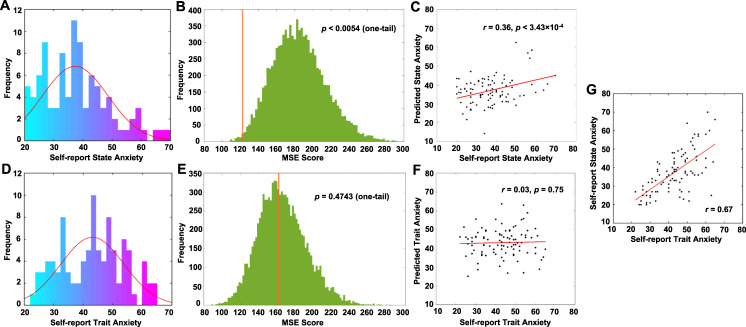
Distribution of anxiety scores and performance of the prediction model. **A** Frequency distribution of levels of state anxiety. **B** Frequency distribution of the MSE scores for the prediction analysis in state anxiety. **C** Scatter plot for the relationship between self-report scores and predicted scores in state anxiety. **D** Frequency distribution of levels of trait anxiety. **E** Frequency distribution of the MSE scores for the prediction analysis in trait anxiety. **F** Scatter plot for the relationship between self-report scores and predicted scores in trait anxiety. **G** Scatter plot for the relationship between state and trait anxiety.

The models were used to attempt to predict both state and trait anxiety, separately. The scores of state anxiety ranged from 20 to 70 (Fig. 3A). The permutation tests showed that the ridge regression model could significantly predict levels of state anxiety above chance (mean alpha = 3.93, *p* < 0.0054, Fig. 3B). The MSE of our model was 122.04, while the mean MSE of the randomized permutation samples was 184.42. Pearson’s correlation between the predicted state anxiety scores and self-reported state anxiety scores was significantly correlated [*r*_(95)_ = 0.36, *p* < 3.43 × 10^−4^, Fig. 3C]. The validation analysis of different cross-validation schemes showed that the results were very robust (Table 1).

**Table 1:** Results of different fold number of cross-validation.

	Alpha	MSE		*r*	
		MSE-value	*p* Value	*r* Value	*p* Value
4-fold	7.60	120.15	0.0076	0.35	4.26 × 10^–4^
6-fold	5.58	122.75	0.0109	0.34	6.53 × 10^−4^
8-fold	3.93	122.04	0.0054	0.36	3.43 × 10^−4^
12-fold	3.42	124.55	0.0103	0.36	3.50 × 10^−4^
16-fold	5.05	124.23	0.0101	0.35	3.97 × 10^−4^

We also fitted the same model in trait anxiety (range: 22–66; Fig. 3D). The permutation test showed that the model was not significantly predictive of levels of trait anxiety (*p* = 0.474, Fig. 3E). Predicted and self-reported trait anxiety were not significantly correlated [*r*_(95)_ = 0.03, *p* = 0.75, Fig. 3F], though the state anxiety was highly correlated with those of trait anxiety (*r*_(95)_ = 0.67, *p* < 1.46 × 10^−13^, Fig. 3G). To test whether the current predictive model is specific to the state rather than trait anxiety, we regressed state anxiety on trait anxiety. Then we used the connectivity-based model to predict the residuals. Results showed that the correlation between predictive scores and residuals was marginally significant (*r* = 0.20, *p* = 0.051), suggesting that the current predictive model is largely associated with state anxiety, independent of trait anxiety.

### RSFC contributions to the prediction of state anxiety

**Fig. 4:**
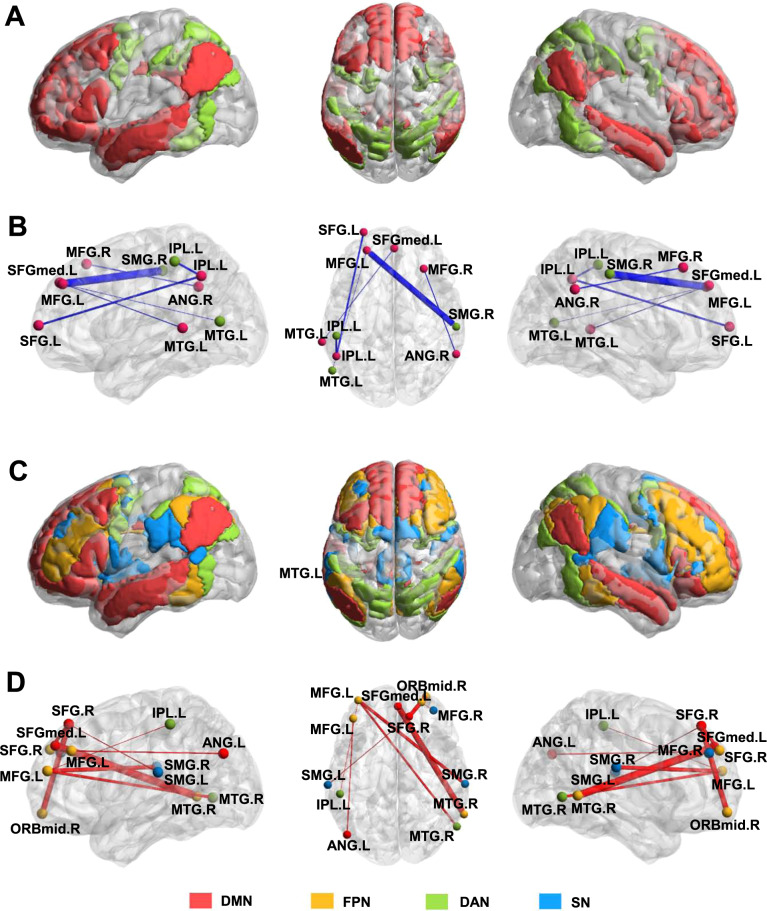
Contributing RSFC in the prediction of state anxiety. **A** The definition of nodes in the default mode network (DMN) and frontal-parietal network (FPN). **B** Brain regions showed negatively weighted connectivity in the DMN and FPN. **C** The definition of nodes in the DMN, FPN, dorsal attention network (DAN), and salience network (SN). D Brain regions showed positively weighted connectivity in the DMN, FPN, DAN, and SN.

**Fig. 5:**
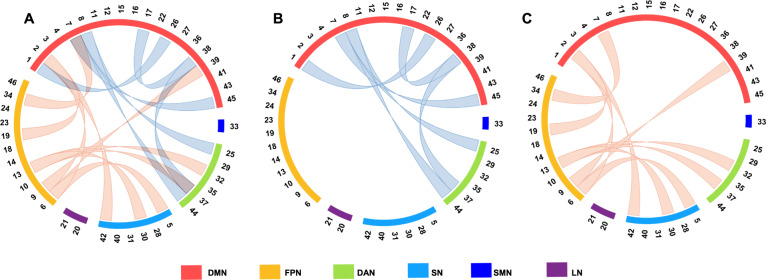
Connectivity patterns of the six contributing networks. Patterns of **A** both negatively and positively weighted connectivity. **B** Connectivity within the DMN, between the DMN and DAN, negatively predicted levels of state anxiety. **C** Connectivity of the FPN with the DMN, DAN, and SN positively contributed to the prediction of state anxiety. See Fig. 1 for the location for each number of the optode. DMN default mode network, FPN frontoparietal network, DAN dorsal attention network, SN salience network, SMN somatomotor network, LN limbic network.

The bootstrap analysis determined the rsFC significantly contributed to the prediction model of state anxiety (Fig. 4; Fig. 5; Table 2). Generally, the contributing RSFC with negative weights was primarily distributed within DMN and between DMN and DAN (Fig. 4A, B; Fig. 5B), while the contributing RSFC with positive weights was primarily distributed between FPN and DMN, FPN, and DAN, as well as FPN and SN (Fig. 4C, D; Fig. 5C).

**Table 2:** Contributing RSFC in the prediction of state anxiety.

Feature (rsFC)	Seed			Target			Weight	Confidence interval	
	Channel	Region	Network	Channel	Region	Network			
Negative weight	1	MFG.R	DMN	26	ANG.R	DMN	−0.6519	−1.2696	−0.0342
	7	SFGmed.L	DMN	45	MTG.L	DMN	−0.5978	−1.1455	−0.050
	8	MFG.L	DMN	44	MTG.L	DAN	−0.545	−1.0374	−0.0526
	17	SFG.L	DMN	38	IPL.L	DMN	−0.7883	−1.5126	−0.0639
	8	MFG.L	DMN	25	SMG.R	DAN	−1.6234	−3.1666	−0.0802
	37	IPL.L	DAN	38	IPL.L	DMN	−0.7641	−1.514	−0.0142
Positive weight	2	SFG.R	DMN	6	SFG.R	FPN	0.8241	0.0626	1.5856
	7	SFGmed.L	DMN	34	MTG.R	FPN	0.9947	0.0729	1.9165
	13	MFG.L	FPN	30	SMG.R	SN	0.854	0.0615	1.6465
	13	MFG.L	FPN	37	IPL.L	DAN	0.7145	0.0721	1.3569
	5	MFG.R	SN	6	SFG.R	FPN	0.7113	0.0113	1.4114
	2	SFG.R	DMN	19	ORBmid.R	FPN	0.9085	0.015	1.8019
	2	SFG.R	DMN	42	SMG.L	SN	0.7256	0.043	1.4083
	9	MFG.L	FPN	39	ANG.L	DMN	0.7408	0.0284	1.4532
	13	MFG.L	FPN	32	MTG.R	DAN	0.8008	0.0005	1.6011

*L* left, *R* right, *MFG* middle frontal gyrus, *SFGmed* superior frontal gyrus (medial part), *IPL* inferior parietal lobule, *ANG* angular gyrus, *MTG* middle temporal gyrus, *SMG* supramarginal gyrus, *ORBmid* middle frontal gyrus (orbital part), *DMN* default mode network, *DAN* dorsal attention network, *SN* salience network, *FPN* frontoparietal network.

## Discussion

In the current study, we established a connectome-based predictive model of state anxiety based on intrinsic connectivity between cortical networks, including DMN, FPN, DAN, and SN. These findings demonstrate a crucial role of cortical regions in the individualized prediction of state anxiety, extending the classical amygdala-centric model of state anxiety. The state anxiety specific predictive model reveals potential neural underpinnings for the distinction of state-trait anxiety. The prediction of state anxiety suggests prominent contributions of state-dependent connectomic characteristics to the prediction of individual differences.

Our results reveal the intrinsic connectivity of cortical network contributes to the prediction of anxiety state. Although state anxiety has been characterized by hyperactive amygdala while trait anxiety is linked with hypoactive prefrontal top-down control in previous models^18,19^, abundant studies show cortical alterations in state anxiety as well. Increased intrinsic connectivity of the DMN with the insula of the SN has also been observed positively correlated with levels of state anxiety in both youth and adults^49^. Altered activation of the brain areas in the SN during threat monitoring has been linked with the individual differences in state anxiety^50^. State anxiety has also been shown to be associated with responses of the vmPFC to threat^51^. Alterations in cross-frequency coupling of cortical oscillations have also been observed to be associated with state anxiety^52^. While hyperactive amygdala representing hyper-functional stimulus-driven bottom-up processing, cortical alterations may indicate abnormal top-down response to the bottom-up input^20^. Hyperactivation in the amygdala in emotion processing in anxious patients has been shown to be associated with its decoupling with the DMN at rest, the strength of which was negatively correlated with state anxiety, suggesting a dysfunctional inhibition of the DMN on the subcortical amygdala activation in anxiety^53^. Alterations of subcortical–cortical connectivity have been widely shown in both state and trait anxiety^50^ during task-specific cognitive processes^54,55^ and task-nonspecific resting state^56–58^. Taken together, these brain features provide substantial candidates for the current predictive models, raising possibilities for the predictive role of cortical networks in anxiety states.

The model established in the current study is specific for the state rather than trait anxiety. State anxiety has been distinguished from trait anxiety by the definition of temporal and persistent characteristics of anxiety. In the psychometrical model, state anxiety is composed by cognitive worry and autonomic emotional dimensions, whereas trait anxiety consists of four dimensions including social evaluation, physical danger, ambiguous, and daily routines^1^. Although common brain networks between state and trait anxiety have been revealed by functional connectivity study^8^, differentiating neural mechanisms of transient versus sustained anxiety has also been shown in generating and regulating anxiety^22^. While a common neural pathway of the AI in the SN with amygdala has been shown between state and trait anxiety with the specificity that state and trait anxiety are associated with the functional and structural connectivity, respectively^56^. These findings converge to common and distinctive mechanisms between state and trait anxiety, providing a potential schemes for diagnosis and treatment of clinical syndromes of anxiety.

Our work provides evidence for prediction of emotion states, besides prediction of personality trait or psychopathology. The connectivity-based predictive model characterizes individual-specific trait profiles in a way as fingerprint^59^, which has been widely applied to cognitive functions^24,26^, personality trait^28,60^, and clinical disorders^61^. However, extensive evidence also shows indispensable contributions of state-dependent functional architecture to Individualized brain organization^62,63^. Brain state manipulations have been shown to improve the prediction of individual traits by amplifying individual variations in specific state^64^. State-independent cortical–subcortical dysconnectivity has been shown to be fundamental for psychosis prediction and characterization^65^. These results jointly suggest that linking state-dependent functional reorganization with an individual differences would contribute to the explanation of behavioral phenotypes and clinical symptoms.

Potentials imitations of the current study should be noted. State anxiety in the current study could be more or less different from the task-evoked transient anxiety. What we measured was a self-reported temporal anxious state without any specific bottom-up input from task manipulations, which might be relatively tonic in comparison to transient anxiety response to a specific stimulus but relatively phasic compared to stable trait anxiety. Therefore, explanations of the state of anxiety should be cautious here. In addition, the measurement channels in the present study could not cover the whole brain because of limited fNIRS probes. However, the configuration in frontoparietal areas was priori defined in terms of the regions characteristic in anxiety^20^ and the regions with high connectivity variability that predominant in individualized prediction^66^. Last but not least, given the comorbidity of depression with anxiety, there might be potential influences of depression on the current results. Trait anxiety mitigated but did not eliminate, the associations of state anxiety with connectivity. While past work has shown that trait anxiety is associated with depression^67^, it is possible that depression had potential confounding effects on our findings. Given that the current study examined healthy individuals with various levels of state anxiety, future studies are necessary to test the utility of the current predictive model for clinical anxiety.

In conclusion, we demonstrate intrinsic connectivity between cortical networks is specifically predictive of individual anxiety state. These findings shed light on the prediction of emotional states and potential neural mechanisms of trait-state distinction in anxiety, which would have important implications for prognosis and diagnosis of anxiety and anxiety disorders.
